# Improving genomic prediction in wheat with random regression models with genotype‐specific phenology‐driven environmental covariates

**DOI:** 10.1002/tpg2.70247

**Published:** 2026-05-08

**Authors:** Rishap Dhakal, Guillermo Sniadower, Paula Silva, Bettina Lado, Pablo Sandro, Inés Rebollo, Martin Quincke, Julie C. Dawson, Lucia Gutiérrez, Pablo González Barrios

**Affiliations:** ^1^ Department of Plant and Agroecosystem Sciences University of Wisconsin‐Madison Madison Wisconsin USA; ^2^ Facultad de Agronomía Universidad de la República Montevideo Uruguay; ^3^ Instituto Nacional de Investigación Agropecuaria (INIA) Colonia Uruguay; ^4^ Department of Agronomy and Plant Genetics University of Minnesota Saint Paul Minnesota USA; ^5^ Department of Plant Breeding Swedish University of Agricultural Sciences (SLU) Alnarp Sweden

## Abstract

Wheat (*Triticum aestivum* L.), a crucial cereal crop for global food security, faces growing challenges from climate change. Future production requires varieties that are resilient to environmental extremes and fluctuations. The goal of this study was to assess strategies to increase selection response through genomic selection in wheat by integrating genotypic‐specific phenology‐derived environmental covariates (ECs) and random regression models (RRM) in multi‐environment trials. We analyzed phenotypic and genomic data from 1683 genotypes from 2010 to 2020 across 71 environments using 45 ECs derived from vegetative, reproductive, and grain‐filling phenological phases. Seven key ECs were selected via partial least squares regression to model genotype by environment interaction (GEI) and evaluate their integration in three different genomic prediction scenarios (CV0, CV1, and CV2). Genomic best linear unbiased prediction models (GBLUP), GBLUP models with GEI (GBLUP_G × E_) modeled as a factor analytic (FA) model, and RRM were compared for their predictive ability performance. RRM with three ECs outperformed GBLUP achieving 50%–100% higher accuracy in CV1 and CV2. The FA exhibited the highest accuracy overall for CV2 but not for CV1. At least one RRM model improved predictions in >89% of environments when predicting new, un‐phenotyped environments. Integrating ECs into the RRM enhances genomic prediction by effectively capturing the GEI with a limited number of covariates.

## Plain Language Summary

Wheat is one of the world's most important food crops, but unpredictable weather makes it hard to breed varieties that produce high yields every year. Plant breeders need better ways to predict how new wheat plants will perform under different conditions. We studied 4291 wheat cultivars tested in 71 environments over 11 years. By combining genomic information with key weather measurements, particularly temperature during early growth and rainfall during flowering, and by using advanced statistical models, we greatly improved yield predictions. Our approach was 50%–100% more accurate than standard methods and worked better in >89% of the environments. This simple, powerful tool will help breeders develop resilient wheat varieties faster, an essential component of food security as climate change brings more extreme and variable weather.

**Abbreviations**
BLUE: best linear unbiased estimate
BLUP: best linear unbiased prediction
EC: environmental covariate
FA: factor analytic
GBLUP: genomic best linear unbiased prediction
GBLUP_G × E_
: genomic best linear unbiased predictions modeling genotype‐by‐environment interactions
GEI: genotype by environment interaction
INASE: National Institute of Seeds
INIA: National Institute of Agricultural Research
PLS: partial least square

## 1 INTRODUCTION

Wheat (Triticum aestivum L.) is a major cereal crop, providing 20% of global human caloric and protein requirements (Reynolds & Braun, 2022). To meet the future global wheat demand without expanding the pressure on land and prices, wheat yield must increase (Fischer, 2022). Due to climate change and extreme weather variability, the frequent occurrence of abiotic (e.g., drought, heat) and biotic stresses (e.g., disease, insect pests) is expected, affecting wheat yield (Hultgren et al., 2025). In response, breeding programs try to develop resilient varieties that can withstand and recover from stress while maintaining high productivity (Agho et al., 2025). To accomplish this, genotype performance and sensitivity to stresses and environmental variability need to be understood (Neyhart et al., 2025).

When the relative response of genotypes changes across environments, it results in genotype‐by‐environment interaction (GEI; Fehr, 1987). GEI can lead to a change in the variance as well as the ranking of genotypes, and multi‐environmental trials are therefore conducted to study these interactions (Cooper & DeLacy, 1994). Strategies proposed to deal with GEI include ignoring it (when overall GEI is low), reducing it by grouping homogenous sets of environments with homogenous variety rankings and developing varieties within these subregions, and exploiting it by developing locally adapted varieties and modelling GEI to design more effective selection criteria (Bernardo, 2010; Lado et al., 2016). Statistical linear models such as the Finlay–Wilkinson model (Finlay & Wilkinson, 1963), Eberhart and Russell (Eberhart & Russell, 1966), fixed linear‐bilinear regression models such as additive main effect and multiplicative interaction (Gauch, 2013) and genotype and genotype by environment interaction  (Yan et al., 2000) models, and mixed models such as factor analytic (FA) models (Smith et al., 2001) have been used to characterize and model GEI. Over the past three decades, a further extension of these linear–bilinear and mixed models has been done, driven by the availability of large‐scale genomic data that allow a better characterization of the genetic components of GEI and the use of this information in making predictions of genotypic performance (Burgueño et al., 2012; Li & Gutierrez, 2023; Piepho & Blancon, 2023).

Genomic prediction (the use of whole‐genome molecular markers to predict breeding or genotypic values of genotypes) has become a relevant method in the plant breeding community in recent years (Escamilla et al., 2025). Genomic selection (genomic prediction‐based selection) can reduce breeding cycle times and increase selection intensity and accuracy, which may result in more rapid genetic gain than conventional phenotypic‐based selection (Edwards et al., 2019). However, for complex quantitative traits such as yield, which exhibit significant GEI, selection based only on genomic prediction models may not be reliable when GEI is high (Burgueño et al., 2012). Consequently, genomic prediction models incorporating GEI have been proposed (Burgueño et al., 2012; Lado et al., 2016).

Plant breeding datasets are unbalanced by nature as not every genotype is tested in all environments. This is because breeders add new lines for testing each year and drop the poorly performing ones from the trials (Dawson et al., 2013; González‐Barrios et al., 2019). Mixed models have been used to deal with this unbalanced nature of datasets (Henderson, 1975). An extension of mixed models is to incorporate the genomic relationship matrix as a covariance structure among genotypes in the model, resulting in a genomic best linear unbiased prediction (GBLUP) for each genotype (Bernardo, 1994; Habier et al., 2007; Osterman et al., 2024; VanRaden, 2008). GBLUP borrows information from relatives (using the genomic relationship matrix) to predict the mean breeding values of the untested genotypes across the environments (Clark & van der Werf, 2013). The GBLUP model was also extended to incorporate GEI components in what was called the GBLUP_G × E_ (genomic best linear unbiased predictions modeling genotype‐by‐environment interactions) model (Burgueño, 2012; Lado et al., 2016) that borrows information from relatives (using the genomic relationship matrix) as well as genetic correlation among environments (e.g., in the form of correlated trait response). Thus, GBLUP_G × E_ can predict the breeding value of untested genotypes in tested environments (Burgueño, 2012). When borrowing information from different environments, different forms of expectations about the covariance structure among environments can be modeled, including unstructured, compound symmetry, diagonal, and FA structures, among others (Butler et al., 2023). GBLUP_G × E_ FA models are more parsimonious than complex unstructured covariances among environments (Malosetti et al., 2016) and give higher predictive ability (correlation between observed response and predicted value) than other covariance structures (Burgueño et al., 2011; So & Edwards, 2011). In GBLUP_G × E_ FA models, loading factors that maximally explain the covariance among environments are first identified. Later, an additional factor is introduced that captures the variation within each environment (Isik et al., 2017). A major drawback of GBLUP_G × E_ FA models or any structured form of GBLUP_G × E_ models is that they cannot be used to predict the genotype's performance in new environments with an unknown correlation structure to the environments present in the data. Also, GBLUP_G × E_ models do not allow hypotheses to be tested regarding genotypic sensitivity to specific stressors or specific environmental drivers of GEI. FA models can be useful as a point of comparison to models including environmental covariates (ECs) as they are expected to be a best‐case scenario for modeling structured GEI.

A further advancement to GBLUP_G × E_ is the incorporation of ECs into random regression models (RRMs), which regress genotype‐specific responses on each predictor factor (Schaeffer, 2004). This approach enhances the ability to predict the performance of genotypes in un‐phenotyped environments (Rebollo et al., 2023; Tadese et al., 2024; Tolhurst et al., 2022), model the GEI using physiological drivers of plant traits (Monteverde et al., 2019), and understand the genotypic sensitivity to ECs (Mumford et al., 2023). Hundreds of ECs information is available with advancements in remote sensing (Xu, 2016). Research on the use of ECs in RRM for understanding GEI and predictions has therefore grown (Basnet et al., 2019; Costa‐Neto et al., 2021; Jarquín et al., 2014; Monteverde et al., 2019; Neyhart et al., 2022; Rebollo et al., 2023; Tadese et al., 2024, 2025; Tolhurst et al., 2022).

Because of issues such as multicollinearity between ECs, which explain small amounts of variation, as well as large *p* small *n* paradigm (i.e., having a large number of predictors and a small number of observations, *p* > > *n*), the use of ECs in RRM is not straightforward (Heslot et al., 2014). Several studies have shown different approaches for including ECs in RRM models for predictions and understanding GEI. One extension has been with GBLUP_G × E_ models, where all available ECs are used for modeling the correlation between the environments instead of using the observed trait correlations (Jarquín et al., 2014). This allows the prediction of genotypes in new environments. However, an assumption for a single variance for an environment and failure to account for the variance–covariance between ECs limits our ability to draw inferences about the GEI drivers from this model (Rebollo et al., 2023). Heslot et al. (2014), on the other hand, used crop growth models to derive the most significant ECs that affect plant growth and development across growth stages of wheat. Later, they incorporated the most significant ECs into factorial regression models along with markers showing variable effects across environments. Additionally, dimension reduction methods like partial least square (PLS) have also been used to select ECs. PLS combined with variable selection methods allows us to select a few ECs that explain the response variable as well as allowing us to identify GEI drivers (Crossa et al., 1999; Monteverde et al., 2019; Rebollo et al., 2023). Later, selected ECs are integrated in RRM for prediction under different scenarios.

The goal of this study was to evaluate the performance of wheat multi‐environment trials by selecting ECs to enhance genomic prediction through random regression and mixed‐effects model approaches. Specifically, the focus was on identifying ECs impact on grain yield prediction. Our objectives were to (1) assess ECs integration into the RRM to improve the predictive ability of a wheat multi‐environment trial; (2) evaluate the effects of ECs on grain yield by modeling the GEI across cross‐validation scenarios. These findings provide a foundation for developing wheat breeding strategies that leverage environmental data to increase resilience and productivity.

## 2 MATERIALS AND METHODS

### 2.1 Data

#### 2.1.1 Phenotypic evaluations

Yield data were collected from multi‐environment trials across multiple years and locations as part of the National Institute of Agricultural Research (INIA) wheat breeding program and the National Institute of Seeds (INASE) wheat national evaluation trials in Uruguay. A total of 4291 genotypes were evaluated, including 3722 genotypes in INIA trials and 431 genotypes in the INASE evaluation trials, with 138 genotypes evaluated in both programs. Among the 4291 genotypes evaluated, 1683 had both phenotypic and genomic information.

Genotypes in this study were part of three trial stages: preliminary yield trials (PYT), advanced yield trials (AYT), and elite yield trials (EYT) of the INIA program, and later INASE trials, as described by Lado et al. (2016). The trials were distributed across four locations in Uruguay: Dolores (33°31′ S, 58°13′ W, 15 m.a.s.l.), La Estanzuela (34°20′ S, 57°42′ W, 81 m.a.s.l.), Young (32°41′ S, 57°38 W, 85 m.a.s.l.), and Ruta 2 (32°42′ S, 57°38′ W, 95 m.a.s.l.). Within these locations, trials were further categorized by sowing date. The optimal sowing window for wheat in these regions extends from May 15 to June 15 for most genotypes, considering all maturity classes in Uruguay (Hoffman Berasain & Castro Tabó, 2012). Accordingly, three sowing periods were categorized for the trials: pre‐optimal (PRE, sown before May 15), optimal (OPT, sown between May 15 and June 15), and post‐optimal (POS, sown after June 15) periods. Evaluations spanned over 11 years from 2010 to 2020. The combination of year, location, and sowing period was defined as the environment. Initially, this resulted in a total of 102 environments. Afterward, environments with less than 25 genotypes were removed from the dataset, resulting in a total of 71 environments.

The EYT were conducted with a randomized complete block design with three to four replications, while PYT and AYT were conducted as resolvable alpha incomplete block design with two to four replications where 6–10 incomplete blocks were nested within each complete replication. The best linear unbiased estimates (BLUEs) for grain yield for all genotypes in each trial with randomized complete block designs were estimated using the following model:

(1) $$y_{ij}=\mu +g_{i}+r_{j}+\varepsilon_{ij}$$

where $y_{ij}$ is the grain yield of the *i*th genotype in the *j*th block, μ is the overall mean, $g_{i}$ is the fixed effect of the *i*th genotype, $r_{j}$ is the independent and identically distributed random variable of the *j*th block in the trial, with $r_{j}∼$ N(0, $\sigma_{r}^{2}$), $\sigma_{r}^{2}$ is the block variance, and $\varepsilon_{ij}$ is an independent and identically distributed random variable, with $\varepsilon_{ij}∼$ N(0, $\sigma_{\varepsilon }^{2}$), where $\sigma_{\varepsilon }^{2}$ is the residual variance and cov($r_{j}$, $\varepsilon_{ij}$) = 0.

The grain yield BLUEs for all genotypes evaluated in incomplete block designs in each trial were estimated using the following model:

(2) $$y_{ijk}=\mu +g_{i}+r_{j}+\beta_{k\left(j\right)}+\varepsilon_{ijk}$$

where $y_{ijk}$ is the grain yield of the *i*th genotype *in the j*th replication for the *k*th incomplete block, μ is the overall mean, $g_{i}$ is the fixed effect of the *i*th genotype, $r_{j}$ is the independent and identically distributed random variable of the *j*th replication within the trial, with $r_{j}∼$ N(0, $\sigma_{r}^{2}$), $\sigma_{r}^{2}$ is the replication variance, $\beta_{k(j)}$ is the independent and identically distributed random variable of the *k*th incomplete block nested within the *j*th replication within the trial, with $\;\beta_{k}∼$ N(0, $\sigma_{\beta }^{2}$), $\sigma_{\beta }^{2}$ is the incomplete block variance, and $\varepsilon_{ijk}$ is an independent and identically distributed random variable, with $\varepsilon_{ijk}∼$ N(0, $\sigma_{\varepsilon }^{2}$), where $\sigma_{\varepsilon }^{2}$ is the residual variance with cov($r_{j}$, $\beta_{k(j)}$) = 0 and cov($r_{j}$, $\varepsilon_{ij}$) = 0.

Additionally, Cullis’ broad‐sense heritability (Cullis et al., 2006) was estimated for each trial present in each environment, defined as $H^{2}=\frac{1-{\bar{v}}_{\Delta }^{\mathrm{BLUP}}}{2\sigma_{g}^{2}}\;,$ where $\sigma_{g}^{2}$ is the genotypic variance and ${\bar{v}}_{\Delta }^{\mathrm{BLUP}}$ is the average standard error of the genotypic best linear unbiased predictions (BLUPs).

#### 2.1.2 Genomic data

Genomic information for 1683 genotypes from the INIA and INASE cultivar registration trials were obtained by genotyping‐by‐sequencing (GBS), as proposed by Elshire et al. (2011) and modified for wheat by Poland et al. (2012). The TASSEL GBSv2 pipeline (Glaubitz et al., 2014), which uses the Chinese Spring cultivar as reference genome (IWGSC CS RefSeq v1.1, International Wheat Genome Sequencing Consortium [IWGSC], 2018), was used to identify 95,000 single nucleotide polymorphisms (SNPs). Loci with a minor allele frequency of less than 5%, a heterozygosity level above 10%, and more than 80% missing values were discarded from the dataset. Missing data were imputed using BEAGLE 5.4 (Browning et al., 2018). After filtering, 46,768 SNPs were recovered.

#### 2.1.3 Environmental information

Daily environmental data were retrieved for all trials using the *NASA Power* R package (Sparks, 2018). In total, 15 ECs were obtained: cumulative precipitation (PP), cloud coverage (CC), evapotranspiration (ET), number of frost days (FD), maximum mean temperature (T $\bar{\max}$), mean temperature (T $\bar{\mathrm{mean}}$), number of days temperature above 25°C (*T* >25), minimum mean temperature (*T* $\bar{\min}$), number of days minimum temperature below 15°C (*T* <15), number of days minimum temperature below 4°C (*T* <4), photothermal quotient (*Q*, the ratio of solar radiation to mean temperature), relative humidity (RH), solar radiation (SR), thermal amplitude (TA), and wind speed (WS) (Table S1).

Thereafter, daily environmental data were aligned with the growing period of each genotype in each trial from sowing to harvest. Consequently, for each specific genotype in a trial, the start and end dates of each phenological interval were used to construct ECs for the interval. The following phenological intervals were considered: vegetative phase (V, from sowing to the start of the reproductive phase), reproductive phase (R, 20 days before anthesis and 10 days after anthesis), and grain‐filling phase (G, end of the R phase to harvest date). These phenological intervals were established following Fisher and Kohn's (1966) definition of the critical period in wheat as a 20‐day pre‐anthesis and 10‐day post‐anthesis window. This approach was used because the exact transition from vegetative to reproductive was not available, whereas heading date was available for most trials. However, heading dates for 20%–90% of genotypes in 30% of the trials were missing. To address this issue, we fit the following model to predict heading dates for the incomplete observations for each genotype in each maturity class:

(3) $$y_{ijk}=\mu +g_{i}+a_{k}+l_{j}+ga_{ik}+gl_{ij}+\varepsilon_{ijk}$$

where $\;y_{ijk}$ is the days from sowing to heading date for the *i*th genotype in the *j*th location in the *k*th year, *g_i_* is an independent and identically distributed random effect of the *i*th genotype, with $g_{i}∼$ N(0, $\sigma_{g}^{2}$), and $\sigma_{g}^{2}$ as the genotypic variance, $a_{k}$ is an independent and identically distributed random effect of the *k*th year, with $a_{k}∼$ N(0, $\sigma_{a}^{2}$), and $\sigma_{a}^{2}$ as the year variance, $l_{j}$ is an independent and identically distributed random effect of the *j*th location, with $l_{j}∼$ N(0, $\sigma_{l}^{2}$), and $\sigma_{l}^{2}$ as the location variance, $ga_{ik}$ is an independent and identically distributed random effect of the *i*th genotype in the *k*th year, with $ga_{ik}$∼ N (0, $\sigma_{ga}^{2}$), and $\sigma_{ga}^{2}$ as the genotype by year variance, $gl_{ij}$ is an independent and identically distributed random effect of the *i*th genotype in the *j*th location, with $gl_{ij}$∼ N(0, $\sigma_{gl}^{2}$), and $\sigma_{gl}^{2}$ as the genotype by location variance, and $\varepsilon_{ijk}$ is an independent and identically distributed random effect, with $\varepsilon_{ijk}∼$ N(0, $\sigma_{\varepsilon }^{2}$), and $\sigma_{\varepsilon }^{2}$ as the residual variance. The covariance among all random effects is equal to zero.

ECs for a genotype in each phenological interval (vegetative, reproductive, and grain filling) were derived from averages, cumulative sums, and other continuous and discrete variables (depending upon ECs) to represent the environmental conditions experienced during the growing season. For each of these three phases, the same set of 15 ECs was calculated, resulting in a total of 45 ECs per genotype across the entire dataset. We use the first letter of the phenological interval (V, R, and G) in conjunction with the abbreviation for the specific EC to refer to these 45 ECs.

### 2.2 EC selection

We used PLSs to select the top ECs for yield prediction from 45 ECs constructed using the full dataset. PLS was performed using the *pls* package in R (Mevik & Wehrens, 2007). The response matrix for PLS represented the grain yield for each genotype, while the 45 ECs for each genotype were the predictors. The top seven ECs were selected to include in our models following their variable importance in PLS projection (VIP) scores as described in Mehmood et al. (2020). The VIP score was computed using the “plsVarSel” package with the VIP function in R (Mehmood et al., 2012).

Additionally, a follow‐up PLS analysis was conducted following a leave‐one‐environment‐out strategy to avoid data leakage from training to prediction sets and to identify additional ECs that might only be relevant for some sets of environments when a specific environment is dropped out of the model. This way, in addition to the previous ECs, we identified two new ECs that became relevant in explaining grain yield: evapotranspiration at grain filling (GET) and minimum mean temperature at grain filling (GT $\bar{\min}$). Finally, after all ECs failed to improve the predictive ability of a few environments that exhibited terminal frost, the performance of the number of frost days at reproductive (RFD) was also evaluated for predicting those environments. The distribution of selected ECs across environments is presented in Figure S1.

### 2.3 Characterization of GEI

For the GEI characterization, we first estimated the variance components from our whole phenotypic dataset and then divided the environments into high‐yielding and low‐yielding years based on the yield data using the *k*‐means method in R (R core team, 2024). Finally, those groups were characterized based on the top seven ECs selected using a circus plot with *CIRCLIZE* R‐Package (Gu et al., 2014).

Variance components were estimated by fitting the following model:

(4) $$\begin{array}{ccc} & & \;y_{ijkmnop}=\mu +g_{i}+a_{j}+l_{k}+s_{m}+\tau_{n\left(jkm\right)}+\beta_{o\left(jkmn\right)}\\ & & +\;\delta_{p\left(jkmno\right)}+ga_{ij}+gl_{ik}+gs_{im}+gals_{ijkm}+\varepsilon_{ijkmnop} \end{array}$$

where $y_{ijkmnop}$ is the grain yield, μ is the overall mean, $g_{i}$ is an independent and identically distributed random effect of the *i*th genotype, with $g_{i}∼$ N(0, $\sigma_{g}^{2}$), and $\sigma_{g}^{2}$ as the genotypic variance, $a_{j}$ is an independent and identically distributed random effect of the *j*th year, with $a_{j}∼$ N(0, $\sigma_{a}^{2}$), and $\sigma_{a}^{2}$ as the year variance, $l_{k}$ is an independent and identically distributed random effect of the *k*th location, with $l_{k}∼$ N(0, $\sigma_{l}^{2}$), and $\sigma_{l}^{2}$ as the location variance, $s_{m}$ is an independent and identically distributed random effect of the *m*th sowing period, with $s_{m}∼$ N(0, $\sigma_{s}^{2}$), and $\sigma_{s}^{2}$ as the sowing period variance, $\tau_{n(jkm)}$ is an independent and identically distributed random effect of the nth trial, with $\tau_{n(jkm)}∼$ N(0, $\sigma_{\tau }^{2}$), and $\sigma_{\tau }^{2}$ as the trial variance, $\beta_{o(jkmn)}$ is an independent and identically distributed random effect of the *o*th replication, with $\beta_{o(jkmn)}∼$ N(0, $\sigma_{\beta }^{2}$), and $\sigma_{\beta }^{2}$ as the replication variance, $\delta_{p(jkmno)}$ is an independent and identically distributed random effect of the *p*th incomplete block, with $\delta_{p(jkmno)}∼$ N(0, $\sigma_{\beta }^{2}$), and $\sigma_{\delta }^{2}$ as the incomplete block variance, $ga_{ij}$ is an independent and identically distributed random effect of the *i*th genotype in the *j*th year, with $ga_{ij}$∼ N (0, $\sigma_{ga}^{2}$), and $\sigma_{ga}^{2}$ as the genotype by year variance, $gl_{ik}$ is an independent and identically distributed random effect of the ith genotype in the *k*th location, with $gl_{ik}$∼ N(0, $\sigma_{gl}^{2}$), and $\sigma_{gl}^{2}$ as the genotype by location variance, $gs_{im}$ is an independent and identically distributed random effect of the *i*th genotype in the *m*th sowing period, with $gs_{im}$∼ N (0, $\sigma_{gs}^{2}$), and $\sigma_{gs}^{2}$ as the genotype by sowing period variance, $gals_{ijkm}$ is an independent and identically distributed random effect of the *i*th genotype in the *j*th year, *k*th location, and *m*th sowing period, with $gals_{ijkm}$ ∼ N (0, $\sigma_{gals}^{2}$), and $\sigma_{gals}^{2}$ as the genotype by year by location by sowing period variance, and $\varepsilon_{ilkmno}$ is an independent and identically distributed random effect of *εth* residual, with $\varepsilon_{ilkmno}∼$ N(0, $\sigma_{\varepsilon }^{2}$), and $\sigma_{\varepsilon }^{2}$ as the residual variance. The covariance among all random effects is equal to zero.

### 2.4 Genomic predictions

#### 2.4.1 The truth model for genomic prediction

Phenotypic data were first analyzed to obtain genotypic BLUEs for each genotype in each trial via Equations (1) and (2), depending on the experimental design. Each trial is nested in an environment (i.e., location, year, and sowing period combinations) and there are multiple trials by environment. The estimated BLUEs were then used as the response variable following the two‐step approach described by Möhring and Piepho (2009). We fit the following model:

(5) $$\hat{y_{ijk}}=\mu +g_{i}+E_{j}+ge_{ij}+\tau_{k\left(j\right)}+g\tau_{ik\left(j\right)}+\varepsilon_{ijk}$$

where $\hat{y_{ijk}}$ is the vector of the genotypic BLUEs for the *i*th genotype in the *k*th trial nested in the *j*th environment, μ is the overall mean, and *g_i_* is the random effect of the *i*th genotype, where **g** ∼ MVN (0, Σ **_g_**) and Σ **_g _= K** $\sigma_{g}^{2}$. Here, $\sigma_{g}^{2}$ is the genotypic variance, and **K** is the realized genomic relationship matrix calculated using genotypic data following the VanRaden (2008) method in the *ASRgenomics* package (Gezan et al., 2022) in R (R core team, 2024); *E_j_* is the fixed effect of the environment (i.e., location, year, sowing time combination); and *ge_ij_* is the random GEI effect where **ge** ∼ MVN[0, (Σ **_E_** ⨂ Σ **_g_**)] and ⊗ is the Kronecker product. In this model, Σ **_E_** was the environmental variance–covariance matrix modeled by fitting a reduced rank FA model with two factors following Meyer (2009) and Burgueño et al. (2012). The $\tau_{k(j)}$ represents the random trial within environment effect, $\tau_{k(j)}∼$ N(0, **I** $\sigma_{\tau \;}^{2}$), where **I** is the identity matrix and $\sigma_{\tau \;}^{2}$ represents the trial within environment variance, $g\tau_{ik(j)}$ represents the random genotype by trial effect, $g\tau_{\mathrm{ik}(j)}∼$ N(0, $I\sigma_{g\tau e\;}^{2}$), and $\sigma_{\;g\tau e\;}^{2}$ represents the genotype by trial variance. The $\varepsilon_{\mathrm{ijk}}$ is the model random residual where 𝝴 follows a multivariate normal distribution. Here, the two‐stage weighted approach was implemented where a parameterization of the first step's BLUE error variance is used as an initial value to model the error variance in this model. The resulting genotypic BLUPs for each trial were subsequently used as the truth (reference value) in the genomic prediction evaluations.

#### 2.4.2 Model selection

For the genomic prediction model, first, we fitted a baseline model (GBLUP). The following model was used:

(6) $$\hat{y_{ijk}}=\mu +g_{i}+E_{j}+ge_{ij}+\tau_{k\left(j\right)}+\varepsilon_{ijk}$$

where $\hat{y_{ijk}}$ is the vector of the genotypic BLUEs for the *i*th genotype in the *k*th trial nested in the jth environment, μ is the overall mean, $g_{i}$ is the random effect of the *i*th genotype with the same assumption as Equation (5), *E_j_* is the fixed effect of the environment (i.e., location, year, sowing period combination), *ge_ij_* is the random GEI effect, $ge_{ij}∼$ N(0, **I** $\sigma_{ge\;}^{2}$), where **I** is the identity matrix and $\sigma_{ge\;}^{2}$ represents the GEI variance, $\tau_{k(j)}$ is the random trial within environment effect with the same assumption as Equation (5), and $\varepsilon_{ijk}$ is the model random residual where 𝝴 comes from a multivariate normal distribution. Here, the two‐stage weighted approach was implemented where a parameterization of the first step's BLUE error variance was used as an initial value to model the error variance in this model.

Second, we fitted the GBLUP_G × E_ FA model with a reduced rank structure following Meyer (2009) and Burgeño et al. (2012). Model (6) was fitted but with the following change: *ge_ij_* is the random GEI effect where **ge** ∼ MVN[0, (Σ **_E_** ⨂ Σ **_g_**)] and ⊗ is the Kronecker product. In this model, Σ **_E_** was the environmental variance–covariance matrix modeled by fitting a reduced rank FA model with two factors following Meyer (2009) and Burgeño et al. (2012), similar to model (5)

Third, we fitted the RRM using the ECs selected through PLS. We identified the RRM with ECs having better median predictive ability, and a forward stepwise selection method was used to incorporate the subsequent five ECs into the model. The model fitted for it was:

(7)

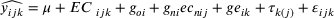

where $\hat{y_{ijk}}$ is the vector of the genotypic BLUEs for the *i*th genotype in the *k*th trial nested within the *j*th environment, μ is the overall mean, $EC_{ijk}$ is the fixed effect of the *e*nvironmental covariates for the *i*th genotype in the *k*th trial of the *j*th environment, $g_{oi}$ is the random intercept (i.e., random additive effect of the *i*th genotype), and $g_{ni}$ is the random slope (i.e., deviation in the yield from $g_{oi}$ for the *i*th genotype for a unit change in $ec_{nij}$). Here, **g** and **gec** ∼ MVN (0, Σ **)**. As the number of ECs was increased, the $\;g_{ni}$ effects and Σ were expanded. For a single EC, Σ =$\;\;\Sigma_{g}$ $⊗\;[\begin{array}{cc} \mathrm{var}(g_{oi}) & \mathrm{cov}(g_{oi},ec_{1})\\ \mathrm{cov}(ec_{1},\;g_{oi})\; & \mathrm{var}(ec1) \end{array}]$ where Σ **g** is the same variance‒covariance matrix explained for the previous Equation (5), ⊗ is the Kronecker product. To make the model comparable with the GBLUP and the GBLUP_G × E_ reduced rank FA model, two terms were also added to the RRM model, where *ge_ij_* is the random genotype‐by‐environment effect with the same assumption as Equation (6) and where $\tau_{k(j)}$ is the random trial‐by‐environment effect with the same assumption as Equation (5). $\varepsilon_{ijk}$ is the model random residual where 𝝴 comes from a multivariate normal distribution. Here, the two‐stage weighted approach was implemented where a parameterization of the first step's BLUEs error variance is used as an initial value to model the error variance in this model. All the linear mixed models were fitted via *ASReml‐R* (Butler, 2020).

#### 2.4.3 Cross‐validation strategies and predictive ability

Alternative schemes were constructed simulating different scenarios a breeder might encounter. The cross‐validation scheme 1 (CV1) represents the prediction of new or un‐phenotyped genotypes in known environments (Bhatta et al., 2020; Burgueño et al., 2012). For a fivefold in CV1, we masked the grain yield BLUEs data of 20% of the genotypes from the dataset at a time to form the validation set. Later, the data of the remaining 80% of the genotypes were used as the training set to predict the masked genotypes’ performance. The cross‐validation scheme 2 (CV2) represents the prediction of genotypes that have been evaluated in some environments but not in others (known genotypes in known environments) (Bhatta et al., 2020; Burgueño et al., 2012). For a fivefold in CV2, we masked grain yield BLUEs data of 20% of the genotypes by environment combinations at a time to form the validation set. Later, the remaining 80% of the data were used as the training set to predict the masked genotype by environment performance. For both CV1 and CV2, we fitted the GBLUP, GBLUP_G × E_ FA2, and RRM models. We employed a fivefold cross‐validation with 50 iterations.

The cross validation 0 (CV0) scheme represents the prediction of genotype performance in a completely new or un‐phenotyped environment (Jarquín et al., 2017) and is sometimes referred to as leave‐one‐environment out. For this, we masked grain yield BLUEs data for all genotypes in an environment to form the validation set. Later, the remaining data were used as the training set to predict the performance of genotypes in the masked environment. We fitted GBLUP and RRM models for CV0 scheme but not the GBLUP_G × E_ FA as this scenario would be unrealistic given that no correlation data would be available.

After each cross‐validation iteration, the predictive ability was calculated as the Pearson's correlation between the predicted values of the masked data and the genotypic values estimated using all data via Equation (5). For the GBLUP and RRM models, this was computed for each genotype in each trial nested within each environment, while for the GBLUP_G × E_ FA model, this was computed for each genotype in each environment. Centering was performed to avoid overestimation due to common environmental effects for both the predicted values for the masked data and the genotypic values calculated via Equation (5) when GBLUP_G × E_ FA was fitted. In CV0, the predictive ability difference was calculated as the deviation in the predictive ability from that of the GBLUP model for each environment to better visualize the gain in the predictive ability when the RRM model with EC was used.

## 3 RESULTS

### 3.1 Dataset characterization

#### 3.1.1 Connectivity and sparsity

**FIGURE 1:**
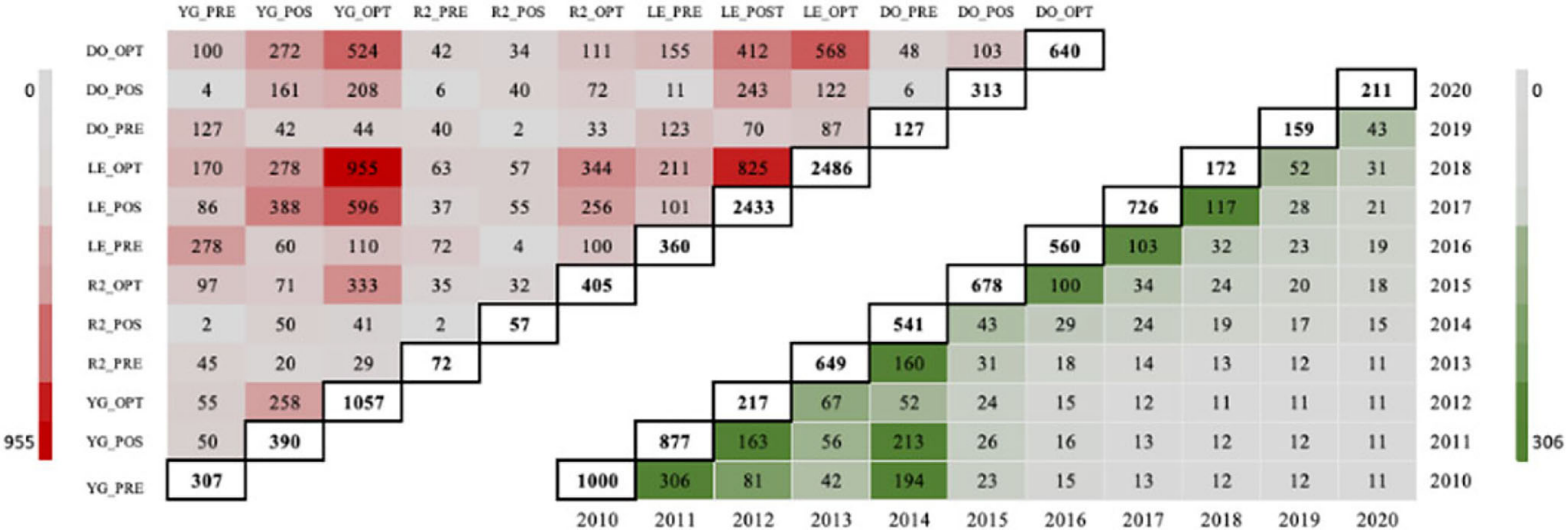
Characterization of the connectivity of genotypes evaluated across sites and years. The upper diagonal heatmap indicates the number of genotypes shared among pairs of sites (location‐sowing intervals) across all years, whereas the lower diagonal displays the number of genotypes evaluated shared among pairs of years. The evaluated locations included Dolores (DO), La Estanzuela (LE), Young (YG), and Ruta 2 (R2) for the pre‐optimal (PRE), optimal (OPT), and post‐optimal (POS) sowing window.

Most of the genotypes were evaluated at La Estanzuela, with fewer present at Young, Ruta 2, and Dolores (Figure 1). Across all the locations, the optimal sowing windows (e.g., OPT) consistently accounted for the largest number of genotypes evaluated. Fewer genotypes were evaluated after 2017.

#### 3.1.2 Heritability

Broad‐sense heritability (*H* ^2^) estimates were consistently high across years and sites (Figure S2), with average *H* ^2^ values exceeding 0.8. The year 2012 presented particularly high heritability levels across trials, whereas 2010, 2015, 2018, and 2020 presented larger variability among sites and lower average *H* ^2^ values. Among the sites, Dolores had the highest average *H* ^2^, whereas Ruta 2 had lower *H* ^2^ values.

#### 3.1.3 GEI and variance component

The largest proportion of the total variance was attributed to year (27.7%) (Table S2). GEI including genotype by year (6.8%), genotype by location (0.5%), genotype by sowing period (0.9%), and the genotype by year by location by sowing period interactions (4.2%), accounted for a substantial portion of the total variance (12.4%). The genotype effect contributed 5.4% to the total variance, which was half of the GEI.

#### 3.1.4 Environment and EC characterization

The top seven ECs selected on the basis of variable importance projection (VIP) PLS scores were cumulative precipitation during the reproductive phase (RPP), maximum mean temperature during the vegetative phase (VT $\bar{\max}$), mean temperature during the vegetative phase (VT $\bar{\mathrm{mean}}$), cumulative precipitation during the vegetative phase (VPP), minimum mean temperature during the vegetative phase (VT $\bar{\min}$), evapotranspiration during the vegetative phase (VET), and number of frost days during the vegetative phase (VFD, Figure S3A). The precipitation at reproductive EC had the highest VIP score, exceeding the other six selected ECs by approximately 20%. While precipitation at reproductive was the only variable linked to the reproductive phase, the remaining six ECs were related to the vegetative phase.

The high‐yielding group included the years 2013, 2015, 2016, 2018, and 2020, with an average yield of 5667 kg ha^−^^1^, whereas the low‐yielding group included the years 2010, 2011, 2012, 2014, 2017, and 2019, with an average yield of 4364 kg ha^−^^1^. High‐yielding environments were generally associated with mild temperature and precipitation, whereas low‐yielding environments, particularly those in 2012, 2014, and 2017, presented higher temperatures and precipitation (Figure S3B). However, the year 2017, which was high‐yielding, was characterized by high precipitation during the vegetative phase.

### 3.2 Genomic predictions

#### 3.2.1 Predictive ability using the CV1 scenario (predicting performance of new or un‐phenotyped genotypes)

**FIGURE 2:**
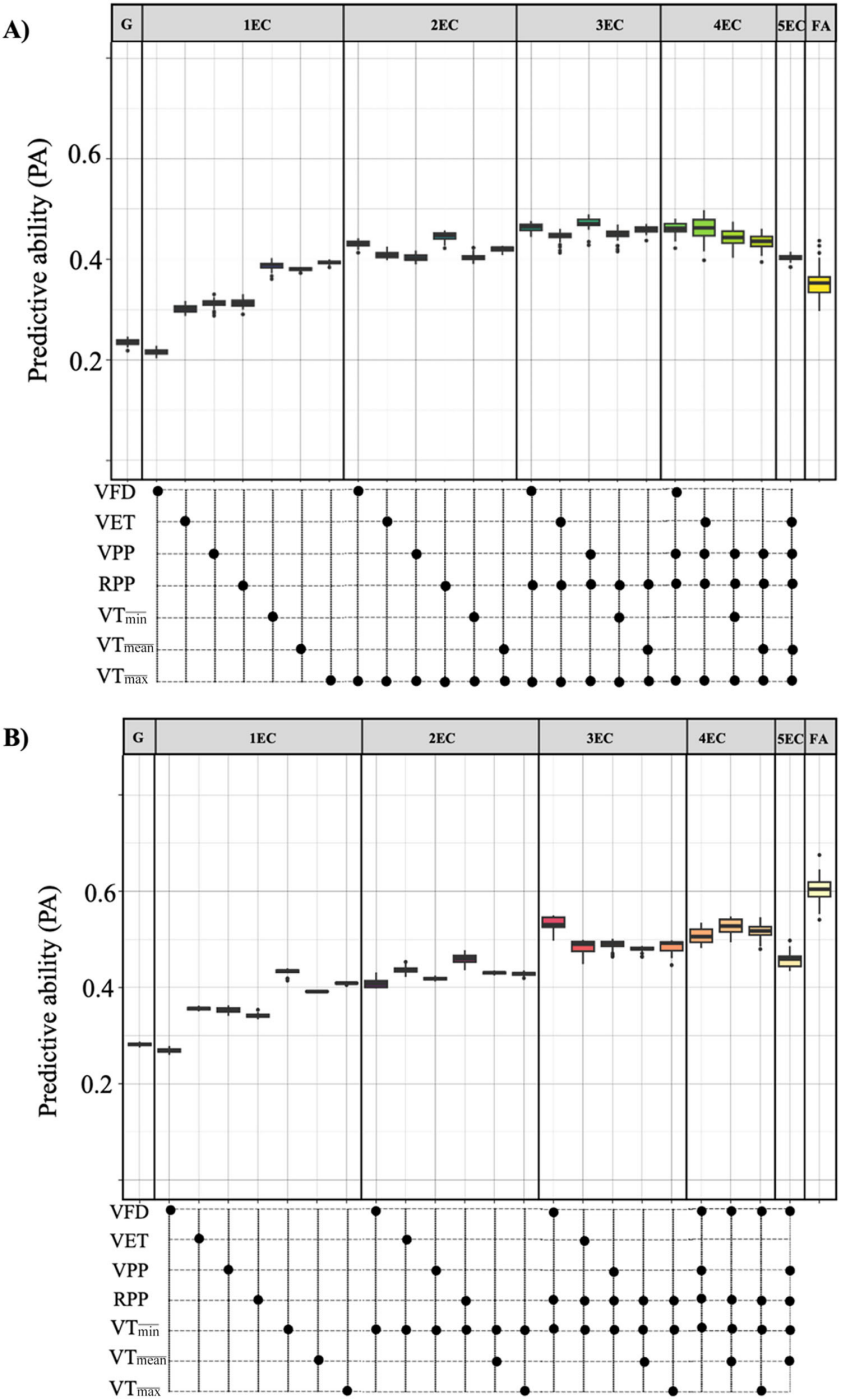
Predictive ability of GBLUP and random regression model (RRM) models under the (A) CV1 and (B) CV2 scenarios. Boxplots represent the distribution of predictive ability values for each grouping: G (GBLUP model), 1EC–5ECs (where EC is environmental covariate) (models incorporating 1 to 5ECs in an RRM framework), and FA (factor analytic variance‒covariance structure of GBLUP_G × E_ models). The lower panel represents the ECs included in each model strategy, categorized by vegetative and reproductive phases: number of frost days at vegetative (VFD), evapotranspiration at vegetative (VET), precipitation at vegetative (VPP) and reproductive (RPP), and minimum (VT $\bar{\min}$), mean (VT $\bar{\mathrm{mean}}$), and maximum (VT $\bar{\max}$) temperature at vegetative.

All RRM models with EC presented higher predictive ability than the GBLUP model except when the number of frost days at vegetative was included alone in the model (Figure 2A). The RRM model with maximum temperature at vegetative was the model with one EC that had the highest median predictive ability (COR = 0.39) with an increase of 70% over the GBLUP model. The best RRM model with two ECs added precipitation at reproductive to the previous model, with a predictive ability of COR = 0.40, which surpassed the GBLUP by 74%. The best RRM with three ECs added precipitation at vegetative to the previous model and had a predictive ability of COR = 0.47, an improvement of 104% over the GBLUP model. The incorporation of a fourth EC, evapotranspiration at vegetative, had a predictive ability of COR = 0.46, a 100% improvement over the GBLUP model. Adding a fifth EC to the best 4EC model did not result in further improvement in the predictive ability beyond the three or four EC RRM model (COR = 40). Finally, the FA model resulted in a predictive ability of COR = 0.35.

Two 5EC combinations (i.e., adding either minimum temperature or number of frost days at the vegetative stage to the best four EC models) could not be assessed due to model convergence failure.

#### 3.2.2 Predictive ability using the CV2 scenario (predicting known genotypes in known environments)

All RRM models outperformed the GBLUP model. The RRM model with minimum temperature at vegetative, precipitation at reproductive, and number of frost days at vegetative was the model that resulted in the highest predictive ability (COR = 0.53) for the three ECs in the RRM, which was 89% greater than that of the GBLUP model. Finally, the FA model yielded a predictive ability value of COR = 0.60. In this scenario, the gap between the best RRM model and the FA model was large, with a predictive ability difference of 0.07, representing a 13% decrease from FA to RRM (Figure 2B).

The RRM with four ECs (i.e., combination of number of frost days, evapotranspiration, minimum temperature, and precipitation at reproductive) failed to converge. Similarly, two 5EC combinations failed to converge: one incorporating number of frost days, evapotranspiration, minimum temperature, precipitation at reproductive, and mean temperature at vegetative, and another replacing evapotranspiration with maximum temperature at vegetative.

#### 3.2.3 Predictive ability using the CV0 scenario (predicting known genotypes in new or un‐phenotyped environments)

**FIGURE 3:**
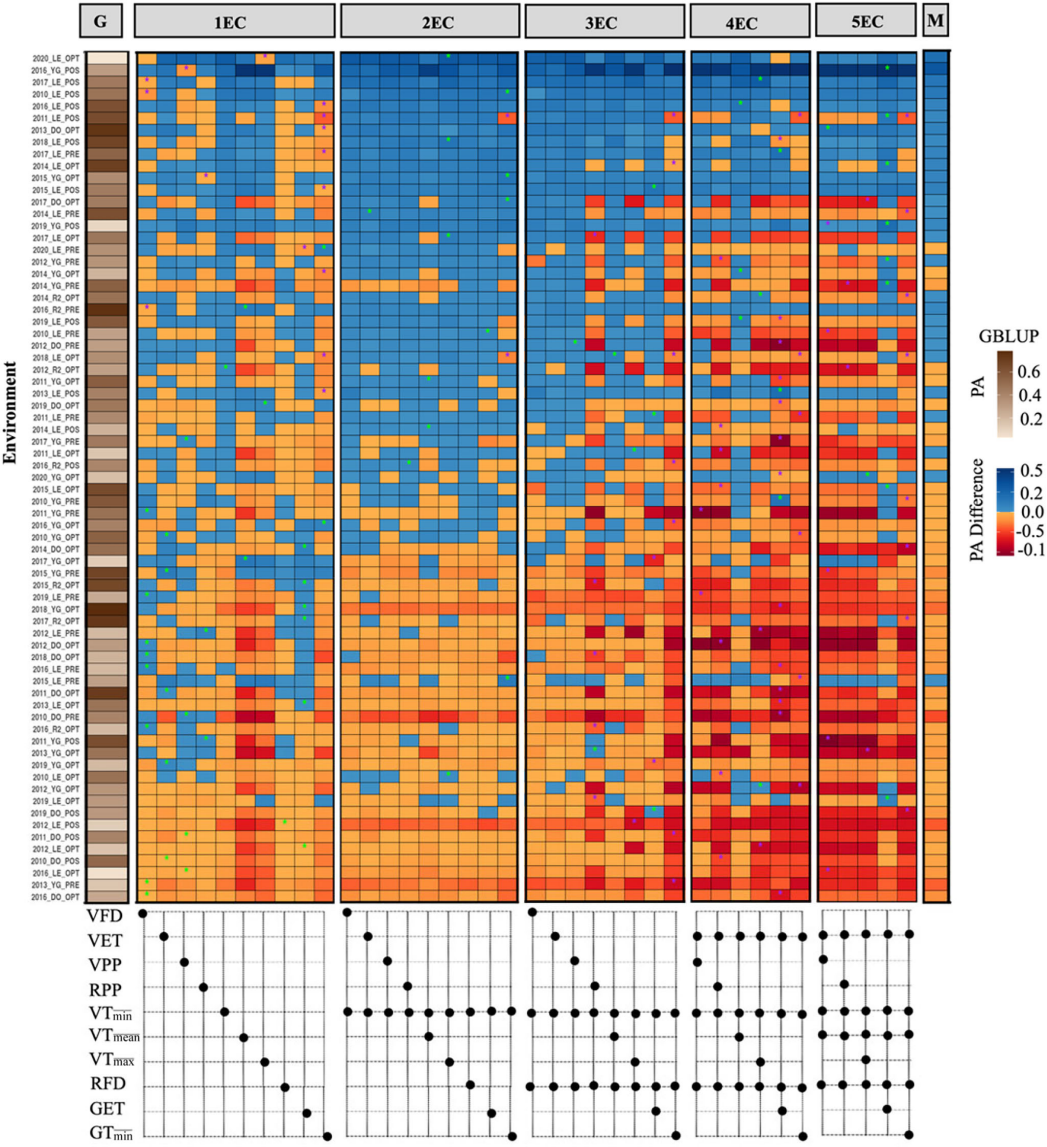
Predictive ability of GBLUP and random regression models (RRM) under CV0 scenarios. The left heatmap shows the predictive ability of the GBLUP model with light brown representing low predictive ability and dark brown representing high predictive ability. The center heatmap shows the predictive ability of each RRM model, expressed as the deviation in predictive ability from the GBLUP model. The right heatmap shows the median of the predictive ability difference across all RRM models for each environment. Blue color in the middle and right panels indicates RRM outperforming GBLUP models for predictive ability, while red colors indicate GBLUP outperforming RRM models. The lower panel represents the environmental covariates (ECs) included in each model strategy categorized by vegetative (V) or reproductive (R) phases: number of frost days at vegetative (VFD), evapotranspiration at vegetative (VET), precipitation at vegetative (VPP) and reproductive (RPP), minimum (VT $\bar{\min}$), mean (VT $\bar{\mathrm{mean}}$), maximum (VT $\bar{\max}$) temperature at vegetative, number of frost days at reproductive (RFD), evapotranspiration at grain filling (GET), and minimum temperature at grain filling (GT $\bar{\min}$). The green stars indicate the best model for an environment, while the purple star represents the worst model for that environment.

The median predictive ability for the GBLUP model across all environments was COR = 0.418 (Figure 3; Table 1). While environment 2012 Dolores at pre‐optimal had the highest predictive ability (COR = 0.78), 2016 Dolores optimal had the lowest predictive ability (COR = 0.02) with the GBLUP model (Figure 3 and Figure S4).

**TABLE 1:** Summary performance of CV0 strategy. GBLUP median predictive ability and random regression model (RRM) model difference in median predictive ability compared to GBLUP, number of environments in which the RRM outperformed the GBLUP model (>GBLUP), number of environments in which the RRM achieved the best or worst performance when fitted with one, two, three, or four environmental covariates.

		Number of times that RRM was		
Model	Median	>GBLUP	best model	worst model
GBLUP	0.418	–	–	–
VFD	−0.005	29	8	3
VET	−0.003	35	5	0
VPP	−0.008	29	4	1
RPP	−0.014	17	2	1
VT $\bar{\min}$	0.009	39	1	0
VT $\bar{\mathrm{mean}}$	−0.144	21	2	0
VT $\bar{\max}$	−0.107	26	1	1
RFD	−0.004	23	1	0
GET	−0.002	35	6	1
GT $\bar{\min}$	−0.152	14	2	8
VT $\bar{\min}$ + VFD	0.007	22	0	0
VT $\bar{\min}$ + VET	0.005	40	1	0
VT $\bar{\min}$+ VPP	0.007	37	0	1
VT $\bar{\min}$+ RPP	0.012	39	1	0
VT $\bar{\min}$ + VT $\bar{\mathrm{mean}}$	−0.009	34	2	0
VT $\bar{\min}$ + VT $\bar{\max}$	0.017	40	3	0
VT $\bar{\min}$ + RFD	0.011	41	0	0
VT $\bar{\min}$ + GET	0.007	37	1	0
VT $\bar{\min}$ + GT $\bar{\min}$	−0.004	33	4	2
VT $\bar{\min}$ + RFD + VFD	−0.003	35	0	0
VT $\bar{\min}$ + RFD + VET	0.009	40	0	0
VT $\bar{\min}$ + RFD + VPP	0.007	40	1	0
VT $\bar{\min}$ + RFD + RPP	−0.187	22	1	5
VT $\bar{\min}$ +RFD +VT $\bar{\mathrm{mean}}$	0.021	39	1	0
VT $\bar{\min}$ + RFD +VT $\bar{\max}$	−0.051	24	1	1
VT $\bar{\min}$ + RFD + GET	−0.003	35	3	2
VT $\bar{\min}$ + RFD + GT $\bar{\min}$	−0.377	11	0	7
VT $\bar{\min}$ + RFD + VET +VPP	−0.097	26	0	2
VT $\bar{\min}$ + RFD + VET +RPP	−0.314	15	0	7
VT $\bar{\min}$ + RFD + VET + VT $\bar{\mathrm{mean}}$	0.010	41	3	0
VT $\bar{\min}$ + RFD + VET + VT $\bar{\max}$	−0.124	21	3	2
VT $\bar{\min}$ + RFD + VET + GET	−0.385	11	3	13
VT $\bar{\min}$ + RFD + VET + GT $\bar{\min}$	−0.288	14	0	6
VT $\bar{\min}$ + RFD + VET + VT $\bar{\mathrm{mean}}$ + VPP	−0.338	17	1	5
VT $\bar{\min}$ + RFD + VET + VT $\bar{\mathrm{mean}}$+RPP	−0.339	18	0	2
VT $\bar{\min}$ + RFD + VET + VT $\bar{\mathrm{mean}}$ + VT $\bar{\max}$	−0.321	16	1	2
VT $\bar{\min}$ + RFD + VET + VT $\bar{\mathrm{mean}}$ + GET	−0.020	33	7	0
VT $\bar{\min}$ + RFD + VET + VT $\bar{\mathrm{mean}}$ + GT $\bar{\min}$	−0.037	12	0	8

The RRM model with minimum temperature at vegetative had the highest predictive ability for 1EC models, improving the GBLUP prediction in 39 out of the 71 environments evaluated (Figure 3; Table 1). The addition of number of frost days at reproductive improved the prediction over the GBLUP in 41 environments (with a median improvement of COR = 0.011). Finally, the other RRM model with the highest predictive ability was achieved via four ECs, where evapotranspiration at vegetative and mean temperature at vegetative was added to the previous model (with minimum temperature at vegetative and number of frost days at reproductive). This model was superior to the GBLUP in 41 environments, with a median improvement of COR = 0.010. Adding minimum temperature at grain filling as the fourth EC to minimum temperature at vegetative, number of frost days at reproductive, and evapotranspiration at vegetative reduced the prediction performance, making this combination the least effective among all the combinations tested (Figure 3). The addition of a fifth EC did not increase the number of environments with improved prediction compared to the previous one (Table 1). Overall, a single model that is, RRM with two or four ECs (minimum temperature at vegetative, number of frost days at reproductive, evapotranspiration at vegetative, mean temperature at vegetative), was able to increase predictive ability in 58% of the environments (Figure 3). For the remaining environments (except for eight environments), models with other EC combinations outperformed the GBLUP model (Figure 3 and Figure S4). The RRM with four EC combinations (number of frost days at reproductive, evapotranspiration at vegetative, mean and minimum temperature at vegetative) could not be assessed due to model convergence failure.

## 4 DISCUSSION

### 4.1 EC selection and their physiological implications

Several alternative ways of incorporating ECs into RRM or genomic prediction models have been suggested (Heslot et al., 2014; Jarquín et al., 2014; Monteverde et al., 2019; Rebollo et al., 2023; Tadese et al., 2024; Tolhurst et al., 2022). However, the best EC inclusion method for RRM is still largely unresolved. In this study, we first derived genotype‐specific phenology‐driven ECs using either observed or predicted heading dates for each genotype in a trial (COR = 0.97) as a proxy to define growth stage boundaries. Later, we used the PLS method for selecting ECs to incorporate into the RRM. ECs selected with PLS have been used in several studies to explore GEI (Crossa et al., 1999; Monteverde et al., 2019; Porker et al., 2020; Rebollo et al., 2023; Vargas et al., 1998, 1999) and have been shown to increase predictive ability when incorporated in RRM (Monteverde et al., 2019; Rebollo et al., 2023). In our study, with a large multi‐environment trials wheat dataset, we were able to select a few ECs from PLS that drive the GEI and to incorporate them in RRM for increasing predictive ability under different scenarios. However, the best model included an EC that was not picked by PLS.

We were also able to associate selected ECs with previously reported wheat stress and their impact on yield and its components. High temperatures during the vegetative phase shorten the phyllochron, causing leaves and tillers to appear faster and accelerate development (Cao & Moss, 1989; Dhakal et al., 2025). When resources are not limited, the first tiller usually appears at the same time as the second to fourth leaves emerge (Alzueta et al., 2012), and tillering is coordinated with leaf appearance afterward (Slafer et al., 2021). However, high temperatures during this time can shift the resource allocation in the plant, decreasing the number of initial spikes per plant. Because a large proportion of tillers die from stem elongation to anthesis (Dreccer et al., 2013), the final number of spikes per plant usually decreases during this period. Later, higher temperatures can divert resources toward leaf and tiller growth, reducing the number of spikelet primordia and resulting in fewer spikelets per spike (Kirby, 1990). This is relevant because wheat is usually sink‐limited during grain filling (Slafer et al., 2023). The shortened growth period may also limit assimilate accumulation, increasing unfilled spikelets, lowering grain weight, and ultimately reducing grain yield (Patrick & Colyvas, 2014). On the other hand, low temperatures during the vegetative phase lengthen the phyllochron, producing fewer leaves and tillers, slower development, and more spikelet primordia (Cao & Moss, 1989). The longer growth period also allows for more assimilate accumulation, supporting better grain filling, higher grain weight, and potentially greater grain yield (Satorre & Slafer, 1999). The cool conditions in the early crop season during this study further highlight the importance of low temperatures during the vegetative and reproductive phases and their impact on yield. In this study, we found that the maximum temperature during the vegetative phase was the most influential EC for the individual genotypic responses to the environment.

Precipitation during the vegetative stage influences early canopy development, tiller formation, and spikelet primordia initiation, with water deficit reducing these components (Satorre & Slafer, 1999) and excessive rainfall causing waterlogging stress (De San Celedonio et al., 2014) and more disease pressure (Riella et al., 2025; Silva et al., 2015). Adequate rainfall early in the season supports vigorous growth, leading to more fertile tillers and higher potential spikelet number (Satorre & Slafer, 1999). Furthermore, during the reproductive stage, insufficient precipitation limits grain set and grain filling, reducing both grain number and grain weight (Satorre & Slafer, 1999). While excess rainfall at this stage can increase disease pressure (e.g., fusarium head blight) (Umpiérrez et al., 2013) and spikelet or grain abortion, ultimately lowering final grain yield (Satorre & Slafer, 1999). Precipitation during the reproductive stage was an influential EC in our study determining genotype‐specific responses to the environment, while precipitation during the vegetative stage had a lower effect.

Higher evapotranspiration in adequate water supply results in enhanced transpiration and photosynthesis, higher biomass accumulation, and more spikelet primordia formation. While excessive evapotranspiration during the vegetative phase under water deficit conditions may cause stress on the shoot apex, reduced assimilate supply, slow spikelet primordia formation, and ultimately fewer spikelets per spike (Fischer & Kohn, 1966). We found that evapotranspiration during the vegetative phase exhibited less of an effect among the ECs in our study.

In this study, we found that the days of frost during vegetative were the least influential among the selected ECs. Later in the development, frost may cause damage or kill the developing shoot apex where spikelet primordia are formed (Barlow et al., 2015). Severe frosts may partially or completely injure the meristem, leading to fewer spikelets or even aborting spike development. Therefore, frost at late development could directly impact the number of spikelets per spike and thus the potential grain number. However, most wheat genotypes are quite tolerant to frost during the early vegetative phase.

### 4.2 Prediction for new genotypes and known genotypes in known environments

We showed that RRM incorporating a few selected ECs increased the predictive ability compared to the GBLUP for both CV1 and CV2 strategies. Previous studies have mainly focused on using all ECs or selected ECs to estimate correlations between environments and leverage this information for predictions (Avagyan et al., 2025; Jarquín et al., 2014; Neyhart et al., 2022). However, such approaches do not account for differences in importance of ECs and do not explicitly model how genotypes respond to the ECs. By using RRM with a small set of selected ECs, we predicted genotype‐specific intercepts and slopes, enabling us to model how each genotype responds to those ECs. This allowed RRM to model both the environmental effects and GEI patterns, improving predictive ability. The predicted intercept and slopes also provide a basis for assessing genotype stability under future climatic variation (Piepho & Blancon, 2023). This becomes crucial in climate change context.

As shown by Monteverde et al. (2019) and Rebollo et al. (2023) in rice, predictive ability in RRM is influenced by the selected ECs and by how effectively those ECs characterize and explain the GEI. We found that ECs related to temperature during the vegetative stage and precipitation during the reproductive stage increased the predictive ability for CV1 and CV2 in our large wheat multi‐environment trials. We also observed a similar pattern in the predictive ability between CV1 and CV2 with one to two EC combinations. This underscores the importance of identifying key EC combinations that consistently drive GEI across environments to allow breeding programs to choose a few RRM models for all their prediction scenarios. The success of the RRMs may be due in part to the use of the crop phenology and deriving the ECs individually for each genotype instead of assuming that all genotypes in a trial experience the same environment.

The GBLUP_G × E_ FA model had the highest predictive ability for the CV2 but not the CV1 scenario. This was expected since the FA model borrows information among known environments through factor loadings and site‐specific variances, making it ideal for predicting missing genotype‐by‐environment combinations when both the genotype and environment have been previously observed (CV2) (A. Smith et al., 2001; A. B. Smith et al., 2005). In contrast, for predicting new, unobserved genotypes (CV1), the RRM is better suited because it models genotypic yield as a continuous function of ECs, utilizing both genomic relationship information and differential genotypic responses across environmental gradients. This allows the RRM to predict genotype‐specific slopes and intercepts for new unobserved genotypes by leveraging their genomic similarity to training genotypes, capturing how each genotype differentially responds to ECs such as temperature and precipitation. Given that no yield data are needed for the RRM, this result shows high potential for the prediction of environments that lack yield data and better predictive ability for predicting new genotypes. Genotypes planted at the same site on the same date are exposed to stresses on the same calendar date but may experience the stress very differently based on their phenological stage at the time. Therefore, in our study, genotype‐specific ECs were derived using phenology information. Although most of the heading date data in our study were predicted, we did not have a completely independent estimation of the phenology stages in our RRMs. However, because heading date (at similar latitudes) is a highly heritable trait that can be predicted with high accuracy (i.e., in our study, the predictive ability for heading date was COR = 0.97), we believe that our results are still robust.

### 4.3 Prediction for new environments

Predicting a genotype yield in new or un‐phenotyped environments is one of the most challenging prediction scenarios (Hu et al., 2025; Malosetti et al., 2016). The only options for prediction in new environments are either to use GBLUP models for predicting average genotypic value or to incorporate ECs to predict environment‐specific genotypic values (Heslot et al., 2014; Piepho, 2022). In this study, we tested this scenario (CV0) by masking one environment in a leave‐one‐environment‐out cross validation scheme, hypothesizing that ECs could improve the predictive ability compared with GBLUP. We were able to predict environments with a predictive ability up to COR = 0.78 with the GBLUP, with a median predictive ability of 0.41, which was remarkable given the challenging task of CV0. The ability of the GBLUP model to predict new, un‐phenotyped environments depends partly on the environment we are trying to predict. GBLUP models will fail, especially when the test environments have strong GEI with the training sets of environments. In our case, for example, the 2016 Dolores optimal environment had the lowest predictive ability in GBLUP (COR = 0.02), with a low genetic correlation with other environments (Figure S4).

By incorporating ECs into the RRM, we were able to increase the predictive ability in 41 out of 71 environments using a single RRM model with minimum temperature at vegetative and number of frost days at reproductive. The predictive ability in the remaining environments, except for eight, was improved by using other RRM models. As expected, the success of the RRM in predicting performance in a new, un‐phenotyped environment (CV0) is environment dependent. When an environment is highly genetically correlated with the rest of the environments and it contributes little to the GEI, the GBLUP model tends to show higher predictive ability, and RRM with ECs provides little or no improvement over the GBLUP (e.g., 2019 Dolores post‐optimal, 2011 Dolores post‐optimal, 2012 La Estanzuela optimal, 2012 La Estanzuela post‐optimal, 2010 Dolores post‐optimal; Figure S4). In contrast, environments contributing strongly to the GEI, those with low to moderate genetic correlations, typically exhibit lower predictive ability under the GBLUP, and in these cases, incorporating ECs into the RRM leads to gains in the predictive ability, presumably because the selected ECs capture some of those GEI patterns (e.g., 2016 La Estanzuela post‐optimal, 2016 Young post‐optimal, 2017 La Estanzuela post‐optimal; Figure S4).

However, we also identified environments that deviated from these patterns; for example, 2013 Young pre‐optimal, 2016 Dolores optimal showed no improvement when using RRM with ECs compared to GBLUP. Several factors could be responsible for these deviations. Climate variability between years is significant, and different ECs or stress factors during the crop growing season may drive yield in a particular year, location, and sowing window. Using PLS to connect yield and ECs in latent space, we were able to select the top seven ECs that explain most of the yield variation (including GEI) across most environments. However, for certain environments, specific ECs during the growing season may be responsible for yield variation; thus, including the top seven ECs did not necessarily improve predictive ability. For example, environments 2013 and 2014 Young pre‐optimal had early maturing genotypes affected by frost during reproductive. Because frost during reproductive was an uncommon event, it was not picked up by the PLS selection. Incorporating it into the RRM, we were able to increase the predictive ability using RRM with EC model compared to the GBLUP for 2014 Young pre‐optimal. However, for the environment 2013 Young pre‐optimal we did not find the increment. For the environment 2013 Young pre‐optimal, there may also be other ECs or factors that are the main drivers of yield variation, which were not included in the top or selected ECs tested in our RRM models. Including those could potentially increase the predictive ability. In contrast, for 2016 Dolores optimal, the lack of improvement with RRM models compared to GBLUP may be due to extreme GEI and very low genetic correlation (e.g., COR = −0.54; Figure S4).

### 4.4 Further remarks

Modeling and predicting yield are complex phenomena and GEI makes them more challenging (Li & Gutierrez, 2023; Peng et al., 2020). Both statistical models (Hoefler et al., 2020; Jarquín et al., 2014; Lado et al., 2016; Malosetti et al., 2016; Millet et al., 2019; Rebollo et al., 2023; Sandro et al., 2024) and crop growth models (Chapman, 2008; Chenu et al., 2017) have been used to understand GEI and make predictions. While statistical models have primarily focused on genotypic prediction in different environments using genomic data and ECs, crop growth models have been used to understand the responses of genotypes given different sources of information (weather, soil, water, and management) combined with genotype‐specific parameters. The true biology of genotypic responses over environmental gradients is often considered to be nonlinear and crop growth models are generally more successful in modeling this nonlinearity (Boer et al., 2007; Hammer et al., 2006; Heslot et al., 2014). However, linear models are sufficient to represent nonlinearity when limited genotypic parametrization information is available or more parsimonious models produce good predictions. In this study, using a linear RRM with few selected ECs, we were able to dissect the ECs driving GEI to predict yield responses with high predictive ability. Furthermore, using only a few ECs in the RRM allowed us to improve predictions in the challenging CV0 scenario for more than 58% of the environments with the best model, and for up to 89% of the environments when other EC combinations were used. This demonstrates the potential of incorporating a small set of ECs into RRM to achieve higher predictive ability for a complex trait like yield.

## 5 CONCLUSIONS

This study highlights the strong interannual variability in wheat yield, which is closely associated with fluctuating environmental conditions across years and evaluation sites. By integrating EC into RRM, we were able to better understand GEI and predict the impact of these factors on yield performance. In particular, the mean maximum and minimum temperature during the vegetative period together with precipitation during the reproductive period were the most influential EC. Additionally, a set of variables associated with the vegetative phase, including precipitation, mean temperature, number of frost days, and evapotranspiration, also influenced GEI for yield.

The use of EC in RRM proved to be an effective strategy across all prediction scenarios (CV1, CV2, and CV0). The inclusion of three ECs resulted in the highest predictive ability for both CV1 and CV2. However, it was achieved with a different set of ECs for both CV1 and CV2. The most predictive ECs for CV0 were environment‐dependent. A single model using two or four ECs increased predictive ability in more than 58% of the environments compared to GBLUP. This suggests that a selected small set of ECs can capture the most relevant environmental information without introducing unnecessary complexity into the models.

In summary, this study demonstrates the relevance of incorporating ECs derived from genotype‐specific crop phenology into genomic prediction frameworks to improve the understanding of GEI and make predictions. However, further research is needed to refine these strategies, particularly for predicting performance in untested environments and under increasingly extreme and frequent climatic conditions. Advancing these approaches will be critical for developing more resilient wheat genotypes and ensuring sustainable production in the face of climate change.

## AUTHOR CONTRIBUTIONS

**Rishap Dhakal**: Conceptualization; data curation; formal analysis; investigation; methodology; software; visualization; writing—original draft; writing—review and editing. **Guillermo Sniadower**: Conceptualization; data curation; formal analysis; investigation; methodology; software; visualization; writing—original draft; writing—review and editing. **Paula Silva**: Data curation; formal analysis; writing—review and editing. **Bettina Lado**: Data curation; writing—review and editing. **Pablo Sandro**: Conceptualization; writing—original draft; writing—review and editing. **Inés Rebollo**: Software; writing—review and editing. **Martin Quincke**: Data curation; writing—review and editing. **Julie C. Dawson**: Writing—review and editing. **Lucia Gutiérrez**: Conceptualization; formal analysis; funding acquisition; investigation; methodology; project administration; resources; supervision; visualization; writing—original draft; writing—review and editing. **Pablo González Barrios**: Conceptualization; data curation; funding acquisition; investigation; methodology; project administration; resources; supervision; visualization; writing—original draft; writing—review and editing.

## CONFLICT OF INTEREST STATEMENT

The authors declare no conflicts of interest.

## Supporting information



Supplemental Material

## Data Availability

The data used and/or analyzed in the current study are available through the Figshare repository, which is available at https://doi.org/10.6084/m9.figshare.30885089.
