# Supplementary material for: Improving genomic prediction in wheat with random regression models with genotype‐specific phenology‐driven environmental covariates
Source: Plant Genome. 2026 May 8;19(2):e70247. doi: 10.1002/tpg2.70247 (PMC13155077; doi:10.1002/tpg2.70247)
Supplement: Supplementary file 1 [file TPG2-19-e70247-s001.docx]

SUPPLEMENTAL MATERIAL

IMPROVING GENOMIC PREDICTION IN WHEAT WITH RANDOM REGRESSION MODELS WITH GENOTYPE SPECIFIC PHENOLOGY DRIVEN ENVIRONMENTAL COVARIATES

Rishap Dhakal^1^, Guillermo Sniadower^2^, Paula Silva^3^, Bettina Lado^2^, Pablo Sandro^1^, Inés Rebollo^4^, Martin Quincke^1^, Julie C. Dawson^1^, Lucia Gutiérrez^5^, Pablo González Barrios^2^.

^1^ Department of Plant and Agroecosystem Sciences, University of Wisconsin-Madison, 1575 Linden Drive, Madison, WI 53706, USA.

^2^ Facultad de Agronomía, Universidad de la República, Av. Garzón, 780, Montevideo 12900, Uruguay.

^3^ Instituto Nacional de Investigación Agropecuaria (INIA), 70006, Colonia, Uruguay.

^4^ Department of Agronomy and Plant Genetics, University of Minnesota, Saint Paul, USA.

^5^ Department of Plant Breeding, Swedish University of Agricultural Sciences (SLU), SE-230 53, Alnarp, Sweden

Corresponding authors: [lucia.gutierrez.chacon@slu.se](mailto:lucia.gutierrez.chacon@slu.se); [pablog@fagro.edu.uy](mailto:pablog@fagro.edu.uy)

**Supplemental Table S1.** Environmental covariates (EC) description.

| **Climatic variable** | **Abbreviation** | **Unit** | **Description** |
| --- | --- | --- | --- |
| Cumulative precipitation | PP | mm | Cumulative precipitation. |
| Cloud coverage | CC | % | The average percentage of cloud cover during the period. |
| Evapotranspiration | ET | MJ/*hr* | The evapotranspiration energy flux at the surface of the earth. |
| Ground Frost | FD | _ | 1 if the temperature was below 0°C at 2 m above ground level and 0 if not. |
| Maximum mean temperature | T$\overline{max}$ | °C | Maximum mean temperature. |
| Maximum temperature above 25°C | T$>25$ | _ | 1 if temperature was above 25°C at 2 m above ground level and 0 if not. |
| Mean temperature | T$\overline{mean}$ | °C | Mean temperature. |
| Minimum mean temperature | T$\overline{min}$ | °C | Minimum mean temperature. |
| Minimum temperature below 15°C | T$<4$ | _ | 1 if the temperature was below 15°C at 2 m above ground level and 0 if not. |
| Minimum temperature below 4°C | T$<15$ | _ | 1 if the temperature was below 4°C at 2 m above ground level and 0 if not. |
| Photothermal quotient | Q | MJ/$m^{2}/^{\circ}C$ | Ratio of solar radiation to mean temperature (Base 0°C). |
| Relative Humidity | RH | % | Mean relative humidity. |
| Solar radiation | SR | $MJ/m^{2}/day$ | Total solar irradiance incident at the surface of the earth under all sky conditions. |
| Thermal amplitude | TA | °C | Daily temperature range. |
| Wind speed | WS | m/s | The maximum hourly wind speed at 2 meters above the surface of the earth. |

**Supplemental Table S2.** Variance components for grain yield (kg ha⁻¹) using data from the complete network of the NWBP database. The relative percentage of the variance components for each source of variability are presented, integrating data from 411

| **Variance Component** | **Proportion (%)** |
| --- | --- |
| Location (L) | 4.7 |
| Year (Y) | 27.7 |
| Sowing period (S) | 2.9 |
| Trial (T) | 27.2 |
| Replication | 6.5 |
| Block | 3.4 |
| Genotype (G) | 5.4 |
| GxY | 6.8 |
| GxL | 0.5 |
| GxS | 0.9 |
| GxYxLxS | 4.2 |
| Residual | 9.1 |
| Total | 100.0 |

trials across 71 environments from 2010-2020.

**Supplemental Figure S1.** Distribution of environmental covariates across environments. The right panel represents the environmental covariates used in the random regression model by vegetative (V) or reproductive (R) or grain filling phase (G) phases: precipitation at vegetative (VPP) and reproductive (RPP), mean (VT$\bar{mean}$) and minimum (VT$\bar{min}$) temperature at vegetative, minimum (GT$\bar{min}$) temperature at grain filling, maximum (VT$\bar{max}$) temperature at vegetative, number of frost days at vegetative (VFD) and reproductive (RFD), evapotranspiration at vegetative (VET) and grain filling (GET). evapotranspiration at vegetative (VET) and grain filling (GET). The environment is sorted from left to right following figure 3.

 **Supplemental Figure S2.** Broad-sense heritability (H²) estimates for grain yield across trials in the NWBP of Uruguay. Boxplots representing the distribution of H² values for each year (2010–2020) and location (Dolores (DO), La Estanzuela (LE), Ruta 2 (R2), and Young (YG)) in Uruguay. Light-colored box plots correspond to individual years, whereas dark-colored box plots represent locations.

**Supplemental Figure S3.** (A) Variable importance in PLS projection (VIP) scores for partial least squares (PLS) analysis of ECs. The scores represent the importance of 45 ECs constructed for different crop growth phases (V: vegetative, R: productive, and G: grain filling). (B) Circus plot showing the seven selected ECs across all the evaluation environments. The environments were grouped into two categories—high-yielding and low-yielding based on year performance. Average values for the seven PLS selected ECs are shown from outer to inner ring: maximum temperature during vegetative phase (VT$\bar{max}$), mean temperature during vegetative phase (VT$\bar{mean}$), minimum temperature during vegetative phase (VT$\bar{min}$), precipitation during reproductive phase (RPP), precipitation during vegetative phase (VPP), Evapotranspiration during vegetative phase (VET), Frost days during vegetative phase (VFD). The color intensity represents higher values of each EC in each environment, with all ECs standardized using the entire dataset.

**Supplemental Figure S4.** Correlation between 50^th^ percentile genetic correlation of each environment and the predictive ability of the GBLUP model under CV0. The gray dots with blue outlines represent the top seven environments in which RRM model showed the highest median increase in predictive ability compared with GBLUP. The gray dots with red outlines represent the bottom four environments in which any RRM model did not increase predictive ability compared with GBLUP. The gray dots with orange outlines represent the next three bottom environments where RRM model, on median, did not increase the predictive ability compared with GBLUP. Plain gray dots represent the remaining environments.
